# Pushout Bond Strength in Coronal Dentin: A Standardization Approach in Comparison to Shear Bond Strength

**DOI:** 10.3390/ma16165667

**Published:** 2023-08-17

**Authors:** Franz-Josef Schröter, Nicoleta Ilie

**Affiliations:** Department of Conservative Dentistry and Periodontology, University Hospital, Ludwig-Maximilians-Universität München, Goethestr. 70, D-80336 Munich, Germany; josi.schroeter@web.de

**Keywords:** bond strength, shear test, pushout test, Weibull, dental adhesives, c-factor

## Abstract

To find an alternative that is closer to clinical reality in terms of cavity geometry and configuration factor, this study investigated the pushout test on in vitro adhesive testing to coronal dentin when compared to the established shear test, both in a standardized approach. For a feasible comparison between both tests, the pushout specimen was adjusted in thickness (1.03 ± 0.05 mm) and cavity diameter (1.42 ± 0.03 mm) to receive a bonding area (4.63 ± 0.26 mm^2^) that matches that of the shear test (4.57 ± 0.13 mm^2^). Though, the configuration factor between both tests differs largely (pushout 1.5 ± 0.08; shear bond 0.20 ± 0.01). The bond strength of five different adhesives (n = 20) was investigated for both tests. The pushout test registered a high number of invalid measurements (30%) due to concomitant dentin fracture during testing. In contrast to the shear test, the pushout test failed to discriminate between different adhesives (*p* = 0.367). Both tests differed largely from each other when comparing adhesive groups. When solely looking at the valid specimens, Weibull modulus reached higher values in the pushout approach. Conclusively, the pushout test in this specific setup does not distinguish as precisely as the shear bond test between different adhesives and needs adaption to be routinely applied in adhesive dentistry.

## 1. Introduction

The factors influencing in vitro bond strength results of dental adhesives involve the used substrate (bovine or human; dentin or enamel), storage condition, specimen’s geometry, film thickness as well as loading condition and modulus of elasticity [1,2]. Since sound human teeth are rarely extracted, in vitro testing focuses on the usage of third molars. They are among the only teeth that are extracted in advance of eruption due to prophylactic reasons. Even though unerupted teeth appear moister when compared to erupted teeth [1], they are at least neither carious nor filled. While morphological changes with increasing tooth age do take place [3], bond strength performance appears to be unaffected by these changes [4].

Aside from the influence of the used substrate, the testing methods vary in the given results. Throughout the development of in vitro bond strength testing on tooth structure, two test methods have been established as the main setups for bond strength testing of dental adhesives, representing 83% of the reported studies in the given review: the micro-tensile and macro-shear test [5]. Depending on the bonding area, a distinction can be made between micro (<3 mm^2^) and macro (>3 mm^2^) tests [6].

Both of those two established methods come with advantages and disadvantages. Among the advantages of the micro-tensile test is obtaining numerous specimens out of one tooth, since sticks usually have a bonding area of 1 mm^2^, instead of the >3 mm^2^ required for macro-shear testing. Further, more adhesive failures are supposed to occur when compared to the shear test, where cohesive failures represent a mentionable problem [1].

On the other hand, fabrication of specimens for micro-tensile bond strength testing is labor intensive and technically demanding, because challenging factors in handling, such as quick dehydration of specimens, further come into place [1]. Cutting the sticks induces stress at the bonding interface, which leads to pre-testing failures during sample preparation, as indicated by the 35.4% pre-testing failures reported when bonding to enamel and 18.2% when bonding to dentin during preparation with a diamond saw [7]. Large criticism arises, as the reporting of pre-testing failures often is sparse [8], with only 30% of papers overall even mentioning pre-testing failures [2]. Further, it is important to accurately report and discern between pre-testing failures and manipulation errors, as pre-testing failures contain failures that occur before tensile testing that are not attributed to human handling, and manipulation errors occur during testing that are attributed to human manipulation [9].

When looking at the shear test, its widespread use can be explained by the plain test protocol, simple specimen preparation and efficient use of substrate, as up to eight specimens can be received out of one tooth. In comparison to the preparation of micro-tensile specimens, tooth cutting takes place prior to adhesive bonding, lowering the irritation of the bonding interface. Also, tooth pieces can be embedded in methacrylate resin in order to improve handling, while micro-tensile sticks remain free of a surrounding substance [10,11]. Meanwhile, both—shear and micro-tensile test—are criticized for the occurrence of cohesive failures, which do not allow exact calculation of bond strength values [12] and are recommended to be excluded from statistical analysis [2]. Amongst others, cohesive failures lead to the scattering of test results, which complicates the comparison between studies [13]. This scattering can be associated with alignment errors [14] and microcracks during cutting [7] in the case of the micro-tensile test, and stress concentration near the loading site due to test configuration and specimen geometry in the case of the shear test [15].

Since these two tests both have varying setups, a detailed description of the used approach or the reference to the applied ISO (International Organization for Standardization) standard needs to be provided in order to establish one generally accepted and conducted testing method for adhesive bond strength testing [2,6,8]. As an alternative testing method, an extrusion (pushout) test for dental purposes was first described in 1970, where a cylinder was pushed out of a disk of dental material in varying plunger diameters to simulate the masticatory cycle, reflecting qualities of clinical relevance [16]. An important factor of clinical relevance in such tests is represented by the configuration factor (c-factor) that describes the ratio of bonded to unbonded surface, as an approximate c-factor of 1.7 represented by the pushout approach is closer to the clinical situation than the 0.2 simulated in shear and tensile tests [17,18]. As polymerization shrinkage stress increases simultaneously with c-factor [17], a pushout approach compared to shear or tensile tests might be better suited for clinical prediction, as in vitro specimens should be subjected to polymerization shrinkage stress prior to bond strength testing [19]. 

Nowadays, the pushout test is not employed as a universal bond strength test and is commonly used to measure retention of fiber posts to root canal dentin [6]. In the few studies in which the test was not only used to determine the bond strength to human root dentin, it displays significantly higher bond strength values in crown dentin when compared to root dentin [20,21]. Endodontically treated roots are cut into slices of up to 2 mm thickness, exposing a small portion of filled root canal in a slightly conical form [22]. A crucial step is the central positioning of the steel plunger on the filling [23], which is used to push out the tested substrate. Critique on the pushout test arises, because of the great variability of the test setup. Variables like plunger size, testing speed, slice thickness and preparation method in terms of borehole size and taper influence results. Further, when testing root canal fillings, the calculation of the true diameter is hardly feasible as root canals are not perfectly round in shape [24]. First attempts to standardize the pushout test as a method to test adhesion to coronal dentin have been made to bovine teeth [25], yet remain to be established. On a positive note, and in contrast to the micro-tensile test, almost no stress at the bonding interface takes place during specimen production, as slices are cut in advance of dentin bonding. Also, there is no need to demount them in any specific matrices’ holder, as in shear tests.

Thus, the present work investigated the applicability of a standardized pushout test setup for adhesive dentistry in comparison to the macro-shear test and shines light on the question of whether the pushout test is equally suited to attain reliable bond strength values of dental adhesives to coronal dentin. The null hypothesis was therefore that with similar bonding surface areas, bonding procedure and test conditions, the outcome of both tests is similar.

## 2. Materials and Methods

Four experimental and one gold-standard self-etch adhesives (Table 1) are used to compare bond strength results of the pushout and shear test. The synthesis and exact compositions of the four experimental adhesives, namely Exp. 1.1–2.2, are addressed elsewhere (submitted paper), as this paper focuses on the comparison of both tests, rather than the influence of the adhesives’ components.

Clearfil SE Bond (CSE; Kuraray Noritake Dental Inc., Kurashiki, Japan) worked as the gold-standard reference and its primer was used for all groups. Primer and adhesive were applied with a microbrush for 20 s each, followed by gentle air drying. Any excess bonding agent was removed with a disposable paper fabric. Light curing was performed for 10 s with a light-curing unit (Bluephase® Style, Ivoclar Vivadent, Schaan, Liechtenstein) with a light-emitting window of 10 mm diameter and an irradiance of 1544 ± 207 mW/cm^2^. A low shrinkage resin-based composite (RBC; Admira Fusion x-tra, AF, VOCO GmbH, Cuxhaven, Germany; LOT 2111693) was applied with gentle pressure through a ball-end plunger to ensure good alignment to dentin. Any excess material was removed, followed by light curing for 20 s.

### 2.1. Pushout Test Specimen Preparation

In total, 47 sound human third molars, stored in 0.2% sodium azide solution at room temperature for no more than three months, were used to produce five groups (n = 20) of test specimens. Teeth were cut with a low-speed diamond saw (IsoMet, Buehler, Lake Bluff, IL, USA) in a vertical direction to produce 1 (±0.1) mm thick slices. Slices were measured with a digital caliper. Tooth slices were continuously stored in distilled water to prevent dehydration of exposed dentin. Specimens were then mounted in a vertical drilling machine (Degussa Dental GmbH, Hanau, Germany) to ensure consistent, perpendicular drilling in the dentin surface. The borehole was positioned in coronal dentin, above the pulp chamber to cut dentinal tubules crosswise and with >1 mm distance to pulp chamber and enamel. The holes were drilled with a parallel chamfer dental diamond burr (Komet Dental, Lemgo, Germany) with a diameter of 1.4 mm and a medium grain size of 107 μm under constant water cooling. Calculation of the bonding area followed the formula for lateral surfaces of cylinders: A = 2 × π × r × h, where A is the lateral area, r the radius and h the height of the cavity. The dimensions were chosen to match the bonding area of the shear test setup. After drilling, specimens underwent bonding procedure and cavity filling. In addition to the regular specimens, seven test specimens of the adhesive Exp. 1.2 without the use of primer were produced. Specimens were then stored in artificial saliva in a thermal oven at 37 °C for 24 h.

### 2.2. Shear Test Specimen Preparation

A total of 20 sound human third molars were used to equally produce five groups (n = 20) of test specimens for the shear test setup. Teeth were cut horizontally to expose coronal dentin, followed by size-dependent sectioning which resulted in a maximum specimen count of eight per tooth. Pieces were embedded in methacrylate resin (Technovit 4004, Kulzer, Hanau, Germany; Powder LOT K010164; Liquid LOT K010108) in a stainless-steel cylinder of 16 mm in diameter. Specimens were randomly allocated to each group; a standardized smear layer was produced with P1200 silicon carbide paper, and they were bonded within 24 h after cutting. Following bonding procedure, specimens were mounted in a matrix holder (Ultradent Products, South Jordan, UT, USA) with a cylindrical split mold (Ultradent Products, South Jordan, UT, USA) for RBC buildups of 2.5 mm in height and 2.4 mm in diameter of the same restorative material following ISO 29022 [26]. Calculation of the bonding area took place by measuring the buildups’ diameters twice followed by calculation of a mean radius r for each specimen. Bonding area calculation then followed the formula of circle areas: A = π × r^2^. Also, seven specimens using adhesive Exp. 1.2 were produced without the usage of primer. Storing condition was equal to the pushout specimens.

### 2.3. Mechanical Testing Methods

The universal testing machine (Z2.5, Zwick/Roell, Ulm, Germany) operated at a crosshead speed of 0.5 mm/min until failure and was used for both test setups.

The pushout test was carried out with a round metal plunger (1.2 mm diameter) on a stainless-steel ring to enable free dislodgement of the filling. The plunger was positioned centrally on the filling and placement was controlled by 2.4× magnifying glasses. The specimen was loaded until failure, i.e., dislodgement of the filling or disruption of the specimen, and the pushout force at failure was measured. Because the test setup resulted in a frequent fracture of tooth slices as it will be shown later, additional specimens were manufactured in order to receive n = 20 specimens eligible for statistical evaluation, which resulted in a total of 142 pushout test specimens.

Shear bond strength testing followed an adaption of ISO 29022 [26] from a notched-edge to a straight-edge chisel. The maximum load at fracture was measured.

Bond strength (BS) was calculated by dividing the maximum load at failure through the individual bonding area of each specimen with the following formula:

BS = F/A

where BS represents the calculated bond strength, F the maximum load at failure and A the individual bonding area.

### 2.4. Microscopic Analysis

**Figure 1:**
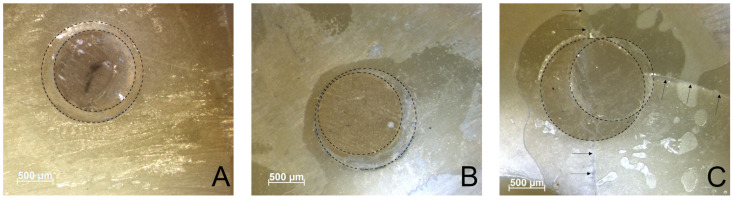
Showcase pictures of the plunger positions central (**A**), margin (**B**) and overlapping (**C**). Cavity circumference is marked by the large, dotted circle, plunger indentation by the smaller, dotted circle. Arrows indicate the visible fracture line within dentin.

Microscopic analysis was performed with a light microscope (Stemi 508, Carl Zeiss Microscopy GmbH, Göttingen, Germany), photographed with a camera extension (Axiocam color 305, Carl Zeiss Microscopy GmbH, Göttingen, Germany) and pictured with AxioVision 4.8.2 computer software. The plunger position was assessed based on the margins of the plunger indentation within the restorative material. Whenever plunger margins were entirely in the restorative material and more than 50 μm distant from dentin, they were classified as central (Figure 1A); when margins were <50 μm away, they were classified as margin (Figure 1B) and lastly, whenever margins intersected dentin for >50 μm (Figure 1C), they were classified as overlapping. Further, light microscopy was used to determine whether a fracture within dentin was visible. If a fracture line was visible in dentin (Figure 1C) on top and on bottom of the specimen, it was classified as invalid and therefore excluded from statistical analysis.

### 2.5. Statistical Analysis

SPSS (IBM SPSS Statistics, Version 28, International Business Machines Cooperation, NY, USA) was used to analyze data. The Shapiro–Wilk test was used to check for normal distribution, and Levene’s test to assess equality of variances. One-way analysis of variances (ANOVA) with Dunnett’s post hoc test compared groups within one test setup. Students t-test for independent variables compared each group with its corresponding group of the other test setup as well as both tests without the use of the adhesives’ primer. A three-way ANOVA was used to evaluate the influence of the parameters’ adhesive, plunger position and dentin fracture causing invalid measurement. Results were considered significant for *p* < 0.05.

Lastly, the reliability of all groups was assessed by Weibull analysis. The model describes the probability of failure for brittle materials at uniform stress with the following formula:

$$Pf=1-exp(-(\frac{\sigma }{\sigma_{0}}{)}^{m})$$

where σ is the measured bond strength, σ_0_ the characteristic strength at probability of failure P_f_(σ_0_) = 0.63 and m the Weibull modulus. The doubled logarithm of this expression $ln[ln(\frac{1}{1-P_{f}})]$ = mln(σ) − mln(σ_0_) results in a straight line. The upward gradient of that line represents m. R^2^ expresses the fit of variances of the observed data towards the projected ideal linear function.

### 2.6. Ethical Approval

No consultation obligation by the institutional ethics committee is needed for this research project. The study was approved under the project number KB 20/032.

## 3. Results

A total of 42 specimens were excluded from the pushout test due to observed dentin fracture after measurement. The number of valid measurements has been upgraded to 100. The mean slice thickness was 1.03 (±0.05) mm, and the mean cavity diameter 1.42 (±0.03) mm. The mean bonding area of the 100 valid specimens was 4.63 (±0.26) mm^2^, while the unbonded area was 3.18 (±0.14) mm^2^. Meanwhile, the mean bonding area of the shear test specimens was 4.57 (±0.13) mm^2^ and 23.51 (±1.55) mm^2^ for the unbonded surface, respectively. Division of the bonded by the unbonded area resulted in a c-factor of 1.5 (±0.08) for the pushout and 0.20 (±0.01) for the shear test specimens. Table 2 displays the mean bond strength, Weibull modulus and R^2^ values for both test setups.

**Figure 2:**
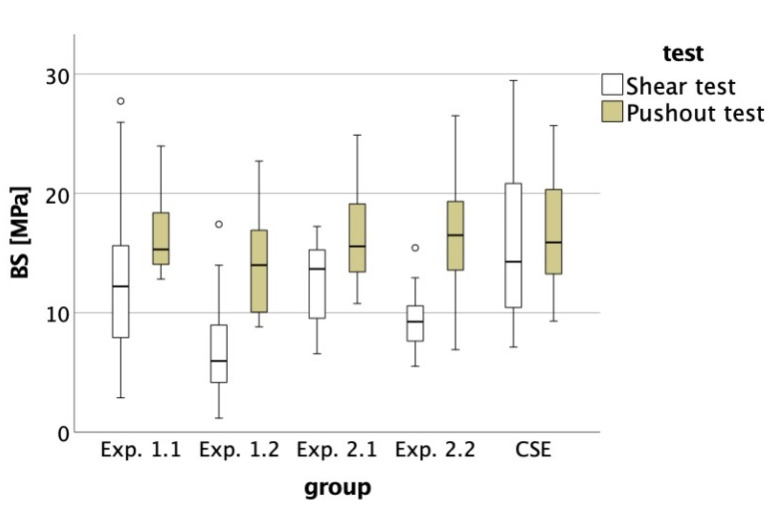
Boxplot for the pushout test and the shear test of the bond strengths (BS) of each group.

The Shapiro–Wilk test confirmed normal distribution for all groups except for Exp. 1.1 (*p* = 0.033) within the pushout test. Data were therefore considered normally distributed. Levene’s test approved equality of variances for the pushout test (*p* = 0.386), but not for the shear test (*p* < 0.001). Thus, ANOVA with Dunnett’s post hoc test was used to check for significant differences within each test setup. While no differences were found in the pushout test (n = 100; *p* = 0.367), differences in the shear test were found (*p* < 0.001). When comparing the two test setups, students t-test showed significant differences between the groups Exp. 1.1 (*p* = 0.02), Exp. 1.2 (*p* < 0.001), Exp. 2.1 (*p* = 0.002) and Exp. 2.2 (*p* < 0.001), but not for CSE (*p* = 0.724). To visualize the differences in bond strength, the boxplot of both tests is provided (Figure 2).

**Figure 3:**
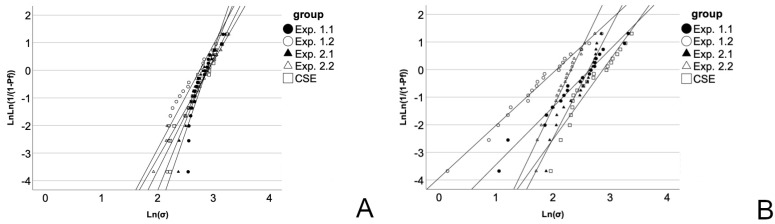
Weibull distribution for the pushout test (**A**) and the shear test (**B**) strength data.

The valid measurements (n = 100) were neither influenced by adhesive group (*p* = 0.858) nor plunger position (*p* = 0.339). Regarding the Weibull modulus, a general trend to higher values was observed in the pushout test. While Exp. 2.2 was inferior to the shear test values, all other groups surpassed the shear test values with CSE, Exp. 1.1 and Exp. 1.2 differing significantly. For the Weibull distribution, see Figure 3.

The results of the Exp. 1.2 specimens without the primer are shown in Table 3. One of the seven pushout specimens was invalid during evaluation, which led to its exclusion. T-test for independent variables showed a significant difference between both tests (*p* < 0.001).

In total, 42 of 142 specimens were declared invalid due to dentin fracture during evaluation. Table 4 shows the error frequency for each group. Of all measurements (n = 142), the used adhesive (*p* = 0.263) and the occurrence of dentin fractures and therefore invalid declaration (*p* = 0.655) had no influence on bond strength, while the plunger position influenced bond strength slightly (η_p_^2^ = 0.057) but significantly (*p* = 0.03).

**Figure 4:**
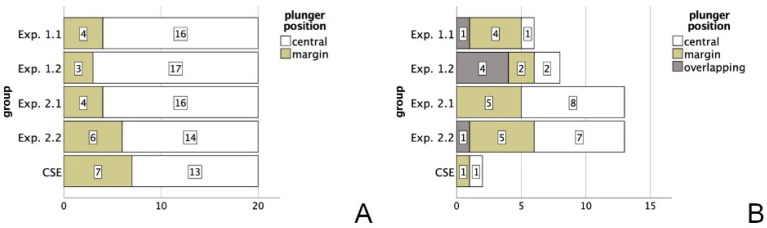
Plunger positions of the valid (**A**) and invalid specimens (**B**).

The plunger position of the valid specimens (n = 100) is displayed in Figure 4A. An ANOVA with only the centered plungers also showed no significant differences within the pushout test (*p* = 0.399). In comparison, Figure 4B shows the plunger position for the 42 invalid specimens, where an overlap was found in 6 cases, marginal position 17 and central position 19 times. The plunger position had no influence on bond strength values of invalid specimens (n = 42; *p* = 0.088).

## 4. Discussion

The aim to draw a scientifically correct comparison between a shear bond and a pushout test needed multiple requirements: Firstly, all specimens were manufactured with equal materials, inside the same laboratory and by the same operator. This renders a comparison between the two tests possible, as a comparison between different laboratories was shown to be difficult [27], since even small differences in local geometry of adhesive interface influence the bond strength results significantly [28]. Secondly, a standardized, reproducible specimen production as stipulated by the literature was conducted [2,6,8] and compared to the already standardized and recognized ISO 29022 method of the shear bond test while ultimately, similar bonded surface areas for both tests were manufactured in order to adequately compare bond strength values as well as their reliability. This resulted in cutting the teeth in vertical slices with a mean thickness of 1.03 (±0.05) mm and by drilling with a chamfer of 1.4 mm diameter in a mean cavity of 1.42 (±0.03) mm diameter, leading to a mean dentin bonding area of 4.63 (±0.26) mm^2^, comparable to the 4.57 (±0.13) mm^2^ area of the shear test.

As polymerization shrinkage of RBCs takes place during light curing, resulting in shrinkage stress [29], specimens for both test methods were produced using the same restorative material and curing conditions. While the used RBC was chosen, because of the low 1.24% polymerization shrinkage [30], shrinkage stress further correlates with the c-factor. The lower the c-factor, the smaller the shrinkage stress. Calculation of the c-factor resulted in a 7.5 times higher value in the pushout test setup compared to the shear test setup, which is in accordance with the results found in other studies [17]. Conclusively, one would assume that shrinkage stresses are higher in the pushout test specimen, which results in imperfect alignment of RBC to the cavity walls ultimately causing quicker failure and thus inferior bond strength values. Though, this can be rejected with the present results as it might be explained by the perfectly parallel cavity walls causing friction during dislodgement that were high enough to overshadow the disadvantages of the higher c-factor.

When addressing bonding areas, Weibull statistics cannot be overlooked, as it is used to determine the reliability of brittle materials by assigning the likelihood of failure to a numeric value, namely the Weibull modulus m. For larger areas, the probability for a critical flaw, such as pores, inclusions or microcracks, to be on the bonding interface is much higher than for smaller areas, resulting in higher bond strengths for smaller areas [31]. In order to minimize the influence of area, bonding areas of both tests closely matched each other. Though, the pushout test found mostly higher m values when compared to the shear test (Table 2), represented by the steeper upward gradient in Figure 3. Four out of five groups exceeded the shear tests’ values, which means that the pushout setting is less susceptible to critical flaws, such as cracks and pores, than the shear test. As non-uniform stress distribution leads to quicker failures of test specimens [2], because the crack propagates from a critical flaw on the bonding interface [31], the higher m in the pushout test might be associated with a more evenly distributed stress, as it was already shown that a homogenous stress distribution when testing glass fiber posts is attainable, revealed by finite element analysis [32]. This leads to a higher m and consequently a higher reliability of measured values in the pushout test, which might be attributed to a less technique-sensitive test protocol compared to the shear test.

As the bonding area was predetermined by the shear bond strength standard ISO 29022, geometry of the pushout test specimen was adapted in slice thickness and cavity diameter. The 1 mm dentin slice thickness was chosen for more than just the reason of matching areas: when comparing literature, slices of usually 1–2 mm thickness are used [24] and 1 mm thickness further allows for a uniform stress distribution [33]. Though, it might be too thin to withstand dentin fracture outside the bonding interface (Figure 1C), which was considered as invalid. Whilst not being considered a pre-testing failure, it can be classified as a manipulation error and is therefore excluded from statistical analysis analogous to the ADM guidelines for micro-tensile testing [9]. As 42 specimens were invalid (30%), an improvement of this test setup’s reliability might be achieved by embedding the specimens in methacrylate resin, as in the shear test, to increase specimen stability. Also, conducting the test under water might help by hindering dehydration of test specimens. Still, testing of aged specimens is challenging, when an identical specimen count per group is desired in order to adequately compare results. As it is criticized in the literature that the reporting of pre-testing failures in micro-tensile testing is often missing [8], this also applies to the pushout test, as fractures outside the adhesive interface as found in the present study (Table 4) are not addressed in any reviews on the study design of pushout tests [24,34,35].

In addition to the thickness of the dentin slices, which determines the height of the cavity, the diameter of the borehole was adjusted to 1.4 mm. This led to an adjustment of the plunger diameter to 1.2 mm for two reasons: when comparing our study design with other protocols, a plunger that is 0.2 mm smaller than the diameter of posts can be used [36] and it furthermore represents 85% of the boreholes’ mean diameter, which in turn should not affect bond strength values [37]. Though, as a plunger size of 70–90% of the canal diameter does not affect bond strength values and smaller strengths are found when the diameter is below 55% [37], the plunger diameter of a smaller size should be chosen to ensure perfect positioning and prevent manipulation errors, while still keeping a standardized diameter, as varying diameters can additionally alter bond strength values [38]. Regarding manipulation errors, the plunger was ideally placed centrally on the filling in order to support uniform stress distribution, which was controlled with magnifying glasses prior to testing. Afterwards, the positioning was controlled microscopically, showing that 136 of the 142 tested specimens had either a central or margin position, while a poor (overlapping) position accounted for only 4% of all measurements. Each one of them resulted in an invalid measurement due to dentin fracture (Figure 4B). Therefore, even though the applied plunger diameter is in the proper range given by the literature [37], a slightly smaller plunger might ease the positioning. The importance of good plunger alignment is also displayed in the small (η_p_^2^ = 0.057), though significant, influence on bond strength values, when considering all measurements, but is usually not addressed in the literature, retrospectively [22,35].

Apart from specimen geometries, another similarity between the two tests was the orientation of the dentinal tubules. Dentinal tubules run radially from the pulp chamber to the dentin surface [39]. During shear test specimen preparation, the horizontal cut above the pulp chamber intersects the tubules nearly perpendicular to their course, which results in the crosswise bonding of tubules. By cutting the tooth vertically and drilling a perpendicular hole in the slice for the pushout test, the dentinal tubules are intersected in the same manner as in the shear test. Although shear bond strength seems to be independent of dentin tubule orientation [40], equal penetration of the tubules during bonding procedure allows for a better comparison of both test methods.

Since the five adhesives did not differ from each other within the pushout setup, a difference when compared to the shear test results seems obvious. All pushout groups but CSE had significantly higher bond strength values than their corresponding shear test groups (Table 2, Figure 2), which leads to the rejection of the null hypothesis. The fact that the differences seen in the shear test do not appear in the pushout test suggests that the adhesive agent is not the decisive factor for bond strength or fracture resistance in this specific setup. Therefore, a few specimens without priming were produced to investigate the influence of flawed application of the adhesive system on bond strength (Table 3). The difference is strikingly obvious, which supports the theory of the measured values’ independency from the adhesives’ performance. Reasonable explanations could be that—as mentioned above—the parallel cavity wall configuration causes friction between RBC and dentin. Usually, the pushout test finds application in endodontological, laboratory trials to test the adhesion of root canal fillings and fiber posts to tooth substrate [34]. Due to the root canal treatment, the canal diameter goes from large to small, resulting in a conical shape of the cavity. Even the conical shape yields friction [37], parallel walls presumably even more. But an exact standard as to which taper needs to be applied has not yet been established, as taper varies largely due to the root canal treatment method, including a taper of 0% [24]. Furthermore, the softer gutta-percha shows lower bond strength (5.86 ± 1.22 MPa) when compared to epoxy resin cones (17.23 ± 4.53 MPa; 16.16 ± 4.73 MPa) and deforms due to compressive stress. Contrarily, stiffer core materials are more resistant to deformation and allow a more linear load profile until dislodgement, resulting in higher bond strength values for stiffer materials that lay in the same range of the bond strengths we found for our materials (Table 2) [37]. This linear load profile might also result in a higher susceptibility to friction, explained by the similar results of all evaluated groups throughout the pushout test.

Summarized, even though the pushout test is closer to reality in terms of c-factor and cavity configuration, it is inferior to the shear test in discerning bond strengths of different adhesives in this specific, standardized setup in vitro. Aside from the perfectly parallel cavity walls, the predetermined bonding area by the shear test as well as the high occurrence of invalid measurements and the slightly too large plunger can be considered as limitations within this study and might influence results, when being changed. Thus, conical cavity walls, alteration in specimen geometry (e.g., thickness, drilling diameter), embedding of specimens in methacrylate resin and testing under water might change the pushout test’s outcome. Also, the testing of aged specimens might be helpful to its long-term applicability. Though, materials are not as susceptible to inherent flaws as in the shear test, shown by the mainly higher Weibull modulus, leading to a higher reliability of measured values in this setup. While more conical cavity walls might help with the problem of friction, the question remains whether it could be better to discern between adhesive groups than the established methods. Lastly, as demanded for the micro-tensile test, pre-testing and manipulation errors within the pushout test must also be accurately reported.

## 5. Conclusions

Within the limitations of the present study, it suggests that the standardized pushout test in this specific setup is inferior to the shear test in measuring adhesives’ bond strength values but is less prone to inherent flaws explained by higher Weibull moduli. Further adjustments are necessary in order to routinely apply the pushout test to adhesive dentistry, including the need to accurately report pre-testing failures and manipulation errors.

## Figures and Tables

**Table 1:** Chemical composition of used materials as provided by the manufacturer.

Name	Composition	LOT
**Exp. 1.1**	bis-GMA, TEGDMA, HEMA, polyacrylic acid, initiators, green tea-extract	-
**Exp. 1.2**	bis-GMA, TEGDMA, HEMA, polyacrylic acid, initiators	-
**Exp. 2.1**	bis-GMA, TEGDMA, HEMA, polyacrylic acid, initiators, tricalcium-phosphate, chitosan, green tea-extract	-
**Exp. 2.2**	bis-GMA, TEGDMA, HEMA, polyacrylic acid, initiators, tricalcium-phosphate, chitosan	-
**CSE**	Primer: 10-MDP, HEMA, DM, initiators Bond: 10-MDP, bis-GMA, HEMA, DM, initiators	2P0372 420696
**AF**	ormocer, 84 wt.% Ba-Al-Si-glass	2111693

Abbreviations: Exp. = experimental adhesive; CSE = Clearfil SE Bond; AF = Admira Fusion x-tra; bis-GMA = bisphenol-A-diglycidyl-methacrylate; TEGDMA = triethylene-glycol-dimethacrylate; HEMA = 2-hydroxyethyldimethylacrylate; 10-MDP = 10-methacryloyloxydecyl-dihydrogenphosphate; DM = dimethylacrylate; ormocer = organically modified ceramic; Ba-Al-Si-glass = barium-aluminum-silicate-glass.

**Table 2:** Bond strength values in MPa and Weibull modulus with 95% confidence interval in brackets, and R^2^ values for both test setups. Superscript letters indicate significant difference within the test setup itself. Asterisk (*) indicates significant differences between the corresponding groups of each test.

	Pushout			Shear		
	BS	m	R^2^	BS	m	R^2^
**Exp. 1.1**	16.5 (15.0; 18.1) *	5.9 (4.61; 7.25)	0.82	12.6 (9.5; 15.6) ^a,c^	2.1 (1.92; 2.25)	0.97
**Exp. 1.2**	14.2 (12.2; 16.2) *	3.8 (3.14; 4.45)	0.88	7.0 (5.1; 8.9) ^b^	1.9 (1.80; 2.01)	0.99
**Exp. 2.1**	16.3 (14.4; 18.1) *	5.0 (4.26; 5.79)	0.91	12.5 (11.0; 14.1) ^a^	4.0 (3.48; 4.48)	0.93
**Exp. 2.2**	16.5 (14.1; 18.4) *	3.5 (3.29; 3.78)	0.98	9.3 (8.1; 10.5) ^b,c^	4.3 (3.81; 4.79)	0.94
**CSE**	16.5 (14.3; 18.7)	4.1 (3.62; 4.49)	0.95	15.9 (12.8; 18.9) ^a^	2.9 (2.44; 3.32)	0.91

Abbreviations: BS = bond strength; m = Weibull modulus; R^2^ = fit of variances to the projected ideal linear function within Weibull statistics; Exp. = experimental adhesive; CSE = Clearfil SE Bond.

**Table 3:** Comparison of bond strength (BS) values (MPa ± standard deviation) of Exp. 1.2 without primer. Asterisk (*) indicates significant differences between both test setups.

	n	BS
Pushout test	6	14.9 (2.3) *
Shear test	7	2.1 (1.2)

**Table 4:** Produced specimens in total and count of errors for each test group.

	Total	Invalid
**Exp. 1.1**	26	6
**Exp. 1.2**	28	8
**Exp. 2.1**	33	13
**Exp. 2.2**	33	13
**CSE**	22	2
**total**	142	42

## Data Availability

The raw data required to reproduce these findings are available upon request.
